# Effects of needle puncturing on re-vascularization and follicle survival in xenotransplanted human ovarian tissue

**DOI:** 10.1186/s12958-023-01081-x

**Published:** 2023-03-20

**Authors:** Hanna Ørnes Olesen, Susanne Elisabeth Pors, Cristina Subiran Adrados, Mette Christa Zeuthen, Linn Salto Mamsen, Anette Tønnes Pedersen, Stine Gry Kristensen

**Affiliations:** 1 Laboratory of Reproductive Biology, The Juliane Marie Centre for Women, Children and Reproduction, Section 5712, University Hospital of Copenhagen, Blegdamsvej 9, 2100 Copenhagen, Denmark; 2 Department of Technology, Faculty of Health, University College Copenhagen, 2100 Copenhagen, Denmark; 3 Fertility Clinic, University Hospital of Copenhagen, Blegdamsvej 9, 2100 Copenhagen, Denmark

**Keywords:** Ovarian tissue cryopreservation, Transplantation, Controlled tissue damage, Needle puncturing, Mechanical injury, Vascularization, Angiogenesis, Follicle survival, Vegf

## Abstract

**Background:**

Ovarian tissue transplantation can restore fertility in young cancer survivors, however the detrimental loss of follicles following transplantation of cryopreserved ovarian tissue is hampering the efficiency of the procedure. This study investigates whether needle puncturing prior to transplantation can enhance revascularization and improve follicle survival in xenotransplanted human ovarian cortex.

**Methods:**

Cryopreserved human ovarian cortex pieces (*N* = 36) from 20 women aged 24–36 years were included. During the thawing process, each piece of tissue was cut in halves; one half serving as the untreated control and the other half was punctured approximately 150–200 times with a 29-gauge needle. The cortex pieces were transplanted subcutaneously to immunodeficient mice for 3, 6 and 10 days (*N* = 8 patients) and for 4 weeks (*N* = 12 patients). After 3, 6 and 10 days, revascularization of the ovarian xenografts were assessed using immunohistochemical detection of CD31 and gene expression of angiogenic factors (*Vegfα*, *Angptl4*, *Ang1*, and *Ang2*), and apoptotic factors (*BCL2* and *BAX*) were performed by qPCR. Follicle density and morphology were evaluated in ovarian xenografts after 4 weeks.

**Results:**

A significant increase in the CD31 positive area in human ovarian xenografts was evident from day 3 to 10, but no significant differences were observed between the needle and control group. The gene expression of *Vegfα* was consistently higher in the needle group compared to control at all three time points, but not statistically significant. The expression of *Ang1* and *Ang2* increased significantly from day 3 to day 10 in the control group (*p* < 0.001, *p* = 0.0023), however, in the needle group this increase was not observed from day 6 to 10 (Ang2 *p* = 0.027). The *BAX*/*BCL2* ratio was similar in the needle and control groups. After 4-weeks xenografting, follicle density (follicles/mm^3^, mean ± SEM) was higher in the needle group (5.18 ± 2.24) compared to control (2.36 ± 0.67) (*p* = 0.208), and a significant lower percentage of necrotic follicles was found in the needle group (19%) compared to control (36%) (*p* = 0.045).

**Conclusions:**

Needle puncturing of human ovarian cortex prior to transplantation had no effect on revascularization of ovarian grafts after 3, 6 and 10 days xenotransplantation. However, needle puncturing did affect angiogenic genes and improved follicle morphology.

## Background

Cryopreservation and autotransplantation of human ovarian cortex tissue has gained ground worldwide as the only fertility preserving option for prepubertal girls and patients in need of immediate cancer treatment [1–4]. Since the first live birth in 2004 [5], the number of live births has now exceeded 200 and live births rates in women transplanted with ovarian tissue range from 20–30% [6–11]. Following ovarian tissue transplantation (OTT) an endocrine recovery of 72–95% has been reported [6]. However, the patients present with consistently low AMH after OTT reflecting the low number of surviving follicles in the ovarian grafts [1, 3, 12–16]. During cryopreservation and thawing less than 20% of the primordial follicles are lost, however, following transplantation 60–80% or more of the follicles are lost during grafting [17–21]. This massive follicle loss observed after OTT is hampering the efficiency of the procedure [22–24], and increasing the follicle survival after OTT could improve reproductive outcomes in transplanted women.

The massive follicle loss in the ovarian grafts is due to ischemia and hypoxia and the injury following reperfusion early in the post-transplantation period [22–24]. Multiple approaches have aimed to reduce the damage caused by hypoxia and ischemia/ reperfusion injury in ovarian tissue with varying results [21, 25–27]. Studies have attempted to accelerate and increase the revascularization of the ovarian graft by the use of vascular endothelial factor (VEGF), basic fibroblast growth factor (bFGF), Er:YAG laser and adipose stem cells to shorten the period of ischemia and to avoid reperfusion failure [21, 28–32]. Revascularization of the ovarian tissue is crucial for its survival and several studies have shown a correlation between improved angiogenesis and higher follicle survival [33, 34], which has made the revascularization process a target of possible optimization of follicle survival after OTT. In cardiovascular surgery, controlled tissue damage has been utilized to induce angiogenesis for decades [35]. Moreover, laser channels has been shown to induce angiogenesis in ischemic animal models [36–41]. In a porcine model tissue damage done by needle punctures was able to induce angiogenesis at the same level as laser treatment [42]. The aim of this study was to study if controlled tissue damage, through mechanical injury using needle puncturing, could induce angiogenesis in xenotransplanted human ovarian tissue and subsequently improve follicle survival and morphology.

## Materials and Methods

### Human ovarian tissue

Donated ovarian cortex tissue from 20 women aged 24–36 years (mean ± SD; 30.6 ± 3.7) was included in this study. The ovarian cortex tissue was cryopreserved for fertility preservation using slow-freezing as previously described [20], at the Laboratory of Reproductive Biology, Copenhagen University Hospital, Denmark between 2001–2011. Indications for fertility preservation for these women were breast cancer (*N* = 10), Hodgkin's lymphoma (*N* = 2), brain cancer (*N* = 1), sarcoma (*N* = 1), benign disease (*N*= 1), and other cancers (*N* = 5).

### Animals

Ten female immunodeficient Naval Medical Research Institute (NMRI)-NUDE mice aged 6–7 weeks were purchased from Taconic, Denmark. Mice were housed in groups, fed pellets and water ad libitum, and kept under controlled 12-h light/12-h dark cycles at 20–22 °C. One week after arrival the mice were anesthetized using Zoletil (Virbac, France), xylazin (Scanvet, Denmark), and butorphanol (Zoetis, New Jersey) before they were ovariectomized to increase endogenous FSH levels. Post-operative analgesia in the form of buprenorphine (Temgesic, Indivior UK Limited, UK) and carprofen (Norodyl, ScanVet, Denmark) was used. Two weeks after ovariectomy either six (4-week study) or eight pieces (3, 6, 10 days study) of human ovarian cortex were transplanted subcutaneously to the back of each mouse. After surgery the mice were single housed for at least 1 week. Euthanasia was performed by cervical dislocation, upon graft retrieval.

### Thawing and needle puncturing

**Fig. 1:**
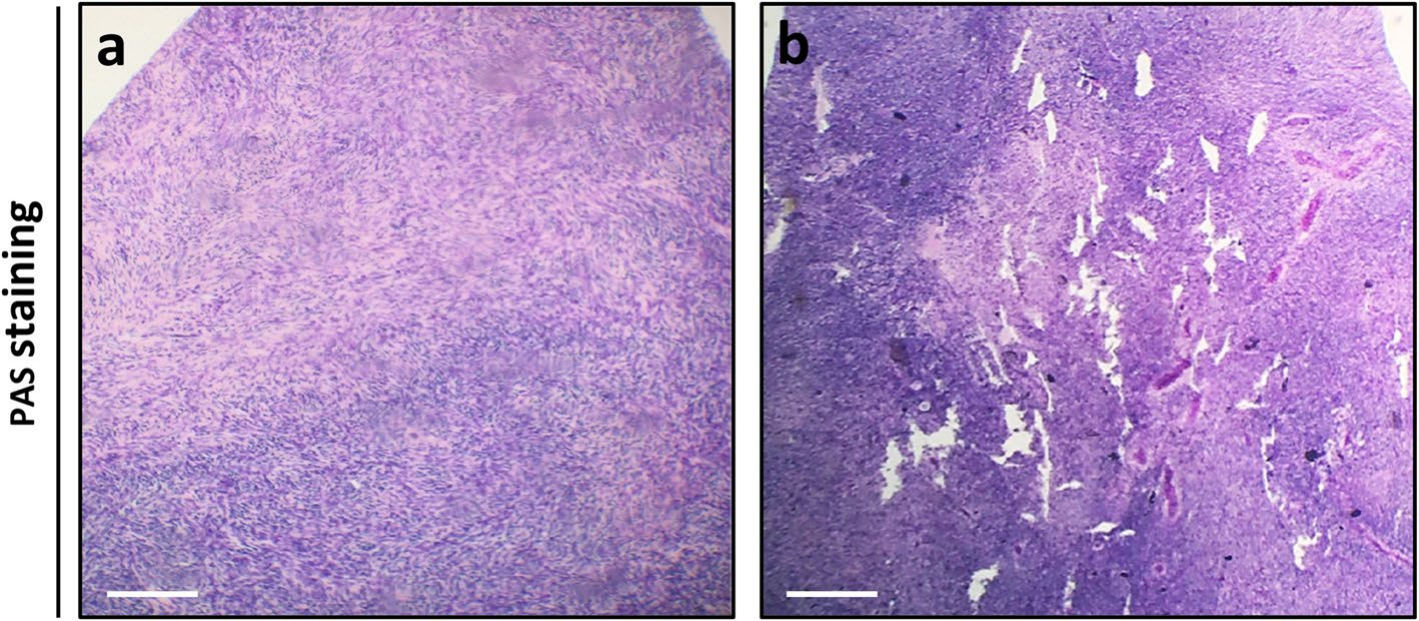
Needle puncturing of human ovarian cortex tissue prior to transplantation. Periodic acid-Shiff (PAS) stained section from cryopreserved and thawed human ovarian cortex tissue. (**a**) Human ovarian cortex tissue with no needle punctures, serving as the untreated control. (**b**) Needle punctured human ovarian cortex, showing holes throughout the tissue. Scale bar = 200 μm

Thirty-six pieces of cryopreserved ovarian tissue was thawed according to standard clinical procedure [43]. Each cortex piece (approximately 5 × 5x2 mm) was cut into two halves, after the initial thawing, where one half functioned as the untreated control and the other half as the piece receiving needle puncturing. The mechanical injury treatment in the form of needle puncturing was performed during the second half of the thawing procedure, without prolonging the thawing process. The treatment was performed by impaling the cortex piece with a 29-gauge needle 150–200 times, to ensure that the whole tissue piece was covered with small puncture wounds, see Fig. 1. A29-gauge needle (0.337 mm) was chosen to inflict small puncture wounds and to avoid removal of tissue during the puncturing. After treatment both treated and untreated tissue pieces were processed through the rest of the thawing process.

### Experimental design and xenotransplantation

**Fig. 2:**
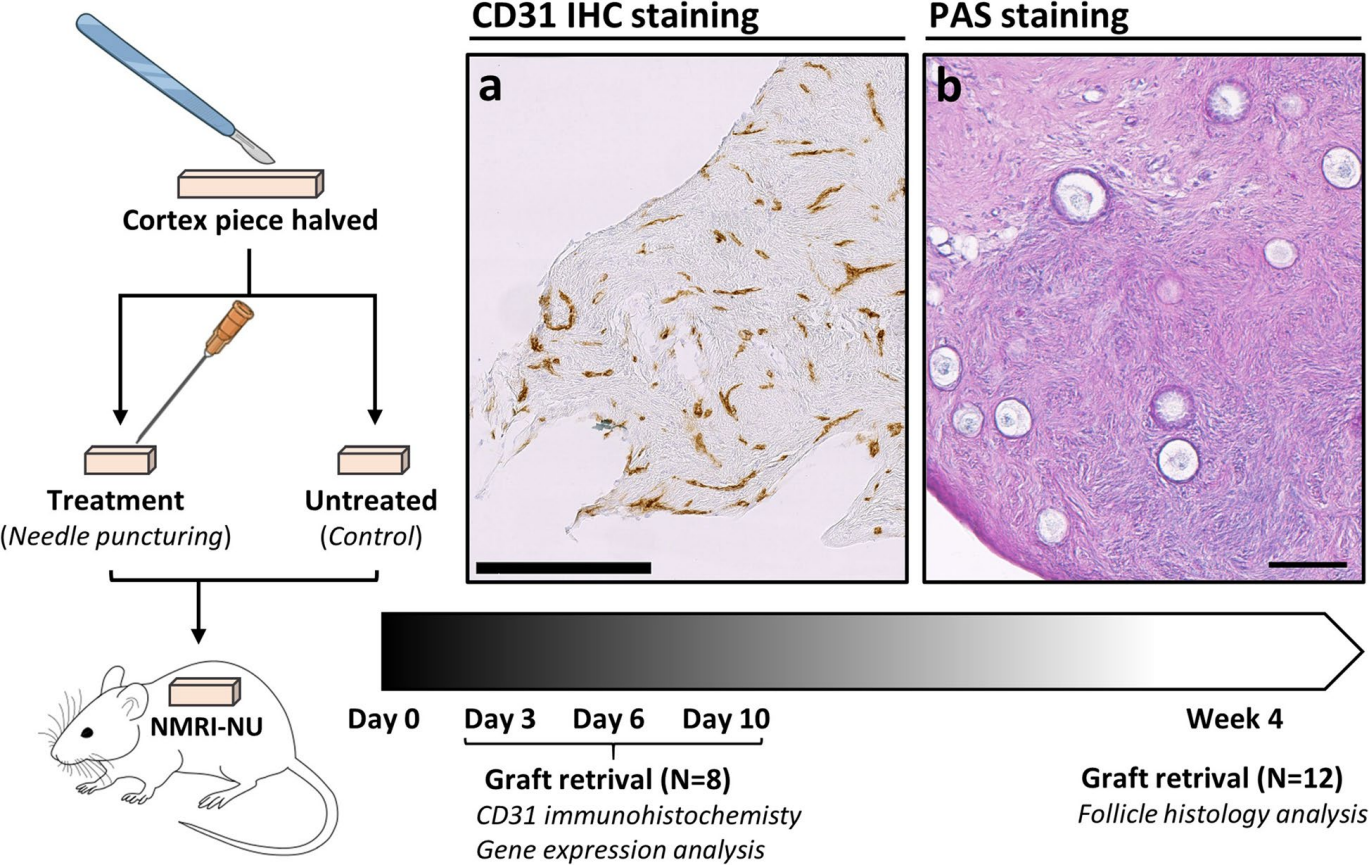
Experimental setup. Cryopreserved ovarian cortex tissue from 20 patients was thawed and cut into two halves with one half receiving needle puncturing and the other half serving as the untreated control. The cortex pieces were xenografted to immunodeficient mice. Grafts was retrieved after 3, 6 and 10 days (*N* = 8) for immunohistochemistry and gene expression analysis, and after 4 weeks for histological analysis of follicle density and morphology. (**a**) CD31 staining after 10 days of xenografting from control group. Scale bar = 250 µm. (**b**) PAS-stained section from 4-week xenografted ovarian tissue showing representative follicles. Scale bar = 100 µm

The experimental design is presented in Fig. 2. After thawing and preparation of the cortex pieces, the pieces were directly transplanted to ovariectomized NMRI-NUDE mice. Two separate xenograft studies were performed in parallel to evaluate the effect of mechanical injury by needle puncturing during short-term (3, 6 and 10 days) and long-term (4 weeks) xenografting compared to an untreated control. Grafts were retrieved after 3, 6 and 10 days to evaluate the critical revascularization window of the ovarian cortex xenograft (*N* = 8; 2 cortex pieces from 8 patients, 8 xenografts per mouse). The 4-week setup was performed for follicle density evaluation because at this time point, atretic follicles has been reabsorbed and only surviving follicles permeate the ovarian xenografts (*N* = 12; 1 cortex piece from 12 patients, 6 xenografts per mouse). Grafts retrieved after short-term xenotransplantation on day 3, 6 and 10, were evaluated for gene expression by quantitative reverse transcription PCR (qRT-PCR) and revascularization by immunohistochemistry (IHC). Follicle counts and morphological assessment was evaluated in the 4-week xenografts.

### Graft retrieval and histological processing

Grafts were dissected from the mice and cleaned for surrounding murine tissue. Grafts retrieved after 3, 6 and 10 days were cut into half; one half for qRT-PCR was snapfrozen and stored at -80ºC, and the other half was fixated in 4% paraformaldehyde (PFA) for IHC analysis. The grafts retrieved after 4 weeks were fixed in Bouin’s solution for histological evaluation. The fixation was done overnight, then dehydrated and embedded in paraffin for sectioning. For IHC the samples were sectioned at 5 μm thickness, and for follicle evaluation the samples were sectioned at 10 μm thickness for Periodic-Acid-Schiff (PAS) staining.

### Quantitative RT-PCR

The human ovarian cortex tissue was homogenized, and RNA-isolation, reverse transcription and qRT-PCR was performed as previously described [26]. In brief, samples were soaked in -80 °C Invitrogen™ RNAlater™-ICE Frozen Tissue Transition Solution (Cat. No. AM7030, ThermoFisher Scientific) overnight and transferred to RLT lysis buffer (Cat. No. 79216, Qiagen) with β-mercaptoethanol. Samples was homogenized using a Qiagen TissueLyser II (Cat. No. 85300) and RNA-isolation was performed with a RNeasy mini kit (Cat. No. 74106, Qiagen) (Silica spin columns: Cat. No 1920–250, EpochLifeScience). Isolated RNA was used to do first-strand cDNA synthesis using the High Capacity cDNA Reverse Transcription Kit (Cat. No. 4368814, Applied Biosystems, ThermoFisher Scientific, Carlsbad, CA, USA) following manufacturer’s instructions. For qRT-PCR analysis TaqMan technology (Applied Biosystems) using TaqMan® fast advanced master mix (Cat. No. 4444964, ThermoFisher Scientific). Predesigned TaqMan® gene expression assays were performed using following genes: Murine Vascular Endothelial Growth Factor Alpha (*Vegfα),*Angiopoietin 1 *(Angpt1),* Angiopoietin 2 (*Angpt2),* Angiopoietin-like 4 *(Angptl4)* and human B-Cell Lymphoma 2 *(BCL2)* and BCL-2 associated X protein* (BAX),* (probe-id: *Vegfα,* Mm00437306_m1; *Angpt1,* Mm00456503_m1; *Angpt2,* Mm00545822_m1; *Angptl4,* Mm00480431_m1; *BCL2*, Hs00608023_m1; *BAX*, Hs00180269_m1). Glyceraldehyde 3-phosphatdehydrogenase (*Gapdh/GAPDH*) was used as endogenous control for both murine and human gene expression assays [44] (probe-id: *GAPDH, Hs*02786624_g1*; Gapdh,* Mm99999915_m1). The cDNA samples were amplified in duplicates using the LightCycler®480 quantitative PCR instrument (Roche, version 1.5.0.39) as previously described [21, 26].

### Immunohistochemical analysis

**Fig. 3:**
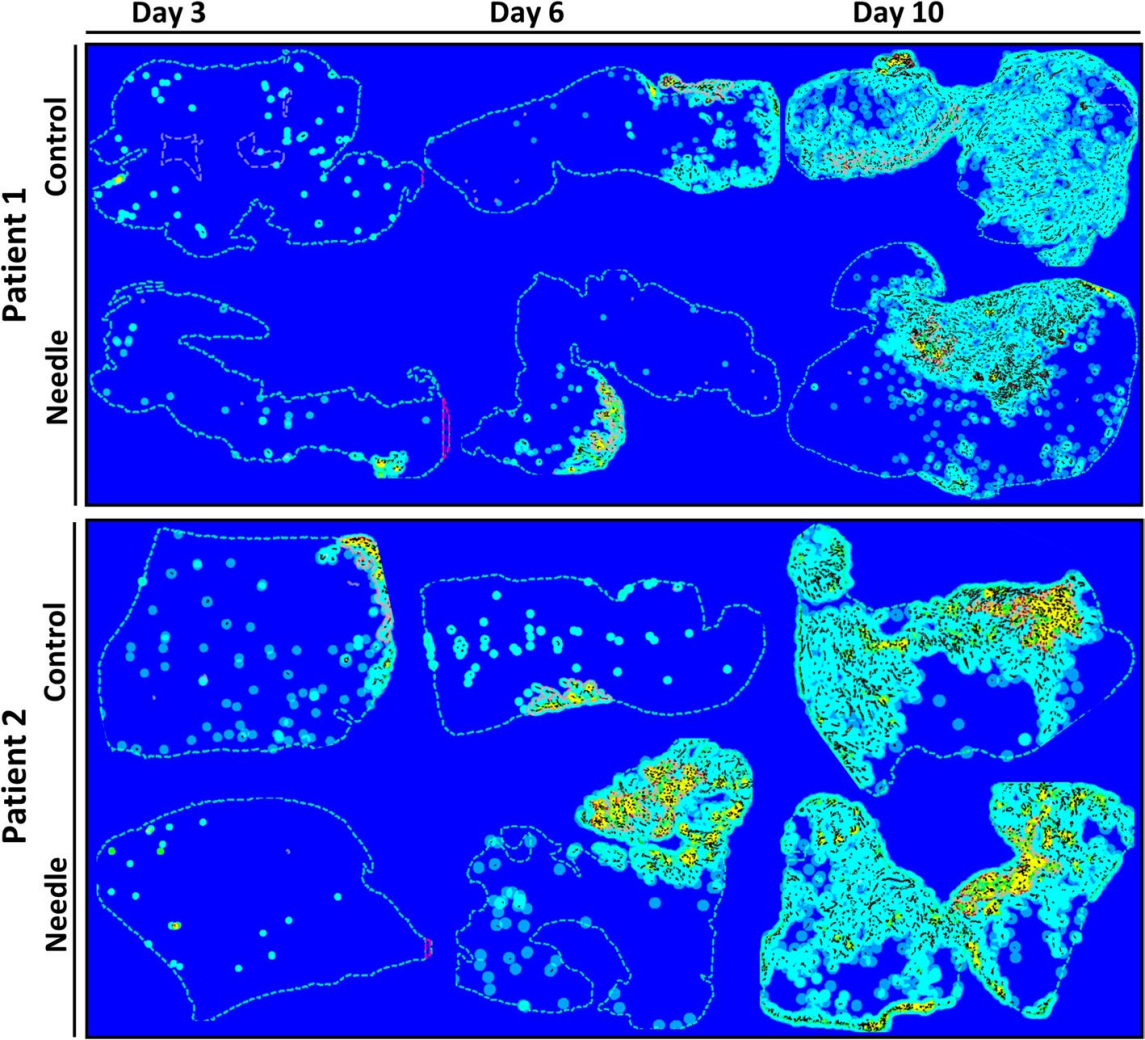
Heatmaps of CD31 stained human ovarian xenografts after 3, 6 and 10 days. Heatmap visualization from needle and control group on day 3, 6 and 10 from two patients

To analyse the revascularization of the xenografts, the endothelial cell marker CD31 was used to visualize murine blood vessels. Four sections from each graft were stained. A murine liver section was included as a positive control, and non-grafted human ovarian tissue was used as a negative control. The IHC analysis was performed as described previously [21, 26]. In brief, sections were de-paraffinized, rehydrated and antigen retrieval was performed using 10 mM sodium citrate (pH 6), following blocking using horse blocking serum (Vector Laboratories, Burlingame, CA, USA) and blocking of endogenous activity with 3% H_2_O_2_. Next, monoclonal anti-rabbit CD31 (Pecam-1) primary antibody (catno.: 77699, Cell Signaling Technology, Herlev, Denmark. Dilution: 1:100) diluted in 1% bovine serum albumin (1:100) was added to the slides and incubated overnight at 4 °C in a humid chamber. The following day the sections were incubated with the secondary antibody (ImmPRESS™ HRP Horse Anti-Rabbit IgG Polymer Detection Kit, Peroxidase, Cat. No. MP-7401, Vector Laboratories, Burlingame, CA, USA), washed and staining was visualized with 3.3′-diaminobenzidine tetrahydrochloride (DAB + Substrate Chromogen System, Dako, Glostrup, Denmark) and briefly dipped in diluted (1:3 water) Mayers Hematoxylin. IgG-negative controls were performed by excluding the primary and only using antibody dilution buffer. Digital pathology using Whole Slide Imaging (WSI) technology and the Visiopharm Author™ module was used to quantify the CD31-positive endothelial area and the number of vessels per mm^2^. The scanning of the slides was performed using the NanoZoomer S360 digital slide scanner with magnification × 40 and one focal point. Scanned WSI files were analysed in the Visiopharm Integrator System (VIS) using the APP via the Visiopharm Author™ module. The APP used has been described earlier [21, 45]. The APP automatically recognized the tissue as the ROI, and afterwards murine host tissue was manually excluded before running the APP. Vessels were subdivided into three categories according to size: micro vessels (< 300 µm^2^), small vessels (300–1000 µm^2^) and large vessels (> 1000–3000 µm^2^). Heatmaps were generated for each section to visualize and highlight the CD31 positive areas in the ovarian tissue xenografts (Fig. 3).

### Follicle density and morphology

Follicle density was evaluated in the xenografts after 4 weeks. Only morphological ‘normal’ follicles with clear nucleus were counted, and follicles were defined and counted as previously described [26]. Primordial follicles were characterized by an oocyte surrounded by a single layer of flat granulosa cells. Primary follicles as oocytes surrounded by a layer of cuboidal granulosa cells, and secondary with two or more layers of cuboidal granulosa cells [46]. Morphologically, follicles were subcategorized as healthy follicles or atretic follicles [47, 48]. For area measurement Olympus BH-2 microscope with Visiopharm Integrator System software (Visiopharm, Hoersholm, Denmark, version 4.6.1779) was used. Density was calculated by the total number of follicles and the volume of the graft based on the area measurements. Follicle morphology was subcategorized (healthy or atretic), percentages from total number of the subcategories was used to visualize the data (Fig. 6).

### Statistical analyses

Statistical analysis was performed using R version 4.2.1. (R Foundation for Statistical Computing, Vienna, Austria) (linear mixed model, multiple comparison Tukey post hoc). Gene expression, CD31 positive area (%), and follicle morphology data was log transformed, before analyzing data. All analyses comparing control and treatment with respect to gene expressions, CD31 positive areas, vessel densities, follicle densities and morphology were analysed with a linear mixed-effects model with patient as a random effect as each woman contributes with more than one sample in the study. Differences between individual groups were analysed using a Tukey post hoc test. *P*-values < 0.05 were considered significant, *p* > 0.10 as tendencies.

## Results

### Revascularization

**Fig. 4:**
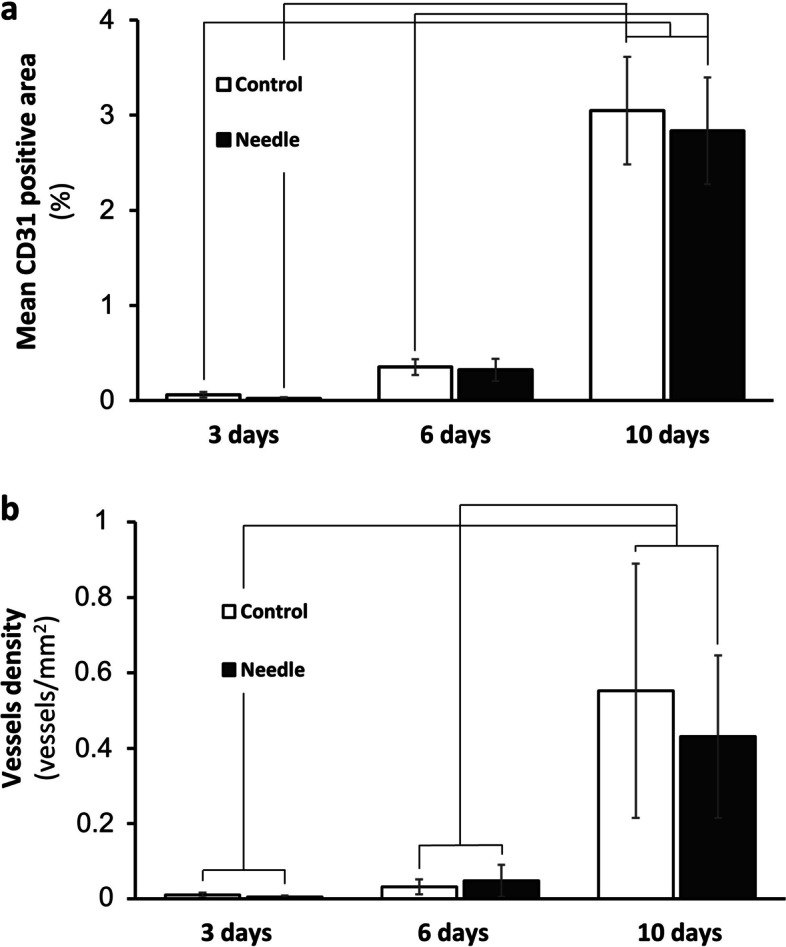
Revascularization of human ovarian xenografts after 3, 6 and 10 days. (**a**) Graph showing mean CD31 positive area of total section area (%) from each timepoint and group (mean ± SEM). (**b**) Graph showing vessel density from each timepoint and group, lines indicate significant difference between timepoints but no treatment effect

The murine CD31 area in the human ovarian cortex tissue was determined after 3, 6 and 10 days of xenografting with or without needle puncturing prior to transplantation. The CD31 positive area was calculated as the percentage of the total section area, and the CD31 density was calculated as the number of vessels per mm^2^. Furthermore, heatmaps was generated to make a visual representation of where the CD31 positive areas was located in the tissue (Fig. 3). There was a significant increase in the CD31 area from day 3 until day 10 with a dramatic increase in the CD31 area between day 6 and day 10 (Fig. 4a). In the 3-day xenografts there was an average of 0.02 ± 0.01% CD31 positive area in the needle group and 0.05 ± 0.02% in the control group. In the 6-day xenografts there was an average of 0.31 ± 0.11% CD31 positive area in the needle group and 0.34 ± 0.08% in the control group. For the 10-day xenografts there was an average of 2.8 ± 0.56% CD31 positive area in the needle group and 3 ± 0.56% in the control group. No significant differences were found in CD31 positive area between the control and needle group.

The vessel density (vessels/mm^2^) on day 3 was 10.26 ± 6.08 vessels/mm^2^ for the control group and 5.62 ± 3.47 vessels/mm^2^ for the needle group (Fig. 4b). On day 6 the vessel density was 32.13 ± 19.74 vessels/mm^2^ for the control group and 48.27 ± 42.7 vessels/mm^2^ for the needle group. The vessel density was 552.9 ± 337.5 vessels/mm^2^ for the control group and 326.4 ± 116.39 vessels/mm^2^ for the needle group on day 10, however, there was no significant difference between needle and control group at any timepoint (*p* = 0.223, *p* = 0.377 and *p* = 0.626). There was a significant increase in vessel density between the different timepoints in both groups (*p* < 0.05).

The APP subdivided the CD31 positive areas into categories according to size: micro vessels (< 300 µm^2^), small vessels (300–1000 µm^2^) and large vessels (> 1000–3000 µm^2^). The distribution of between the subgroups was not different at any timepoint and the main proportion of vessels was micro vessels (97–88%). Table 1 includes the data from CD31 and the distribution of the subgroups.

**Table 1:** Revascularization data after 3, 6 and 10 days grafting

	**3 days**		**6 days**		**10 days**	
	**Control**	**Needle**	**Control**	**Needle**	**Control**	**Needle**
**Vessel density** (vessels/mm^2^)	0.005 ± 0.005	0.003 ± 0.003	0.016 ± 0.016	0.024 ± 0.030	0.276 ± 0.242	0.165 ± 0.099
**Mean CD31 positive area** (%)	0.04 ± 0.039	0.02 ± 0.013	0.35 ± 0.082	0.32 ± 0.118	3.05 ± 0.566	2.84 ± 0.563
**Micro vessels** (%)	94.7	97.8	92.7	88.0	89.9	90.0
**Small vessels** (%)	5.1	2.2	6.5	10.7	8.7	8.4
**Large vessels** (%)	0.2	0.0	0.8	1.3	1.4	1.6

Data presented as densities and percentages ± standard error of mean (SEM)

### Expression of angiogenic and apoptotic genes

**Fig. 5:**
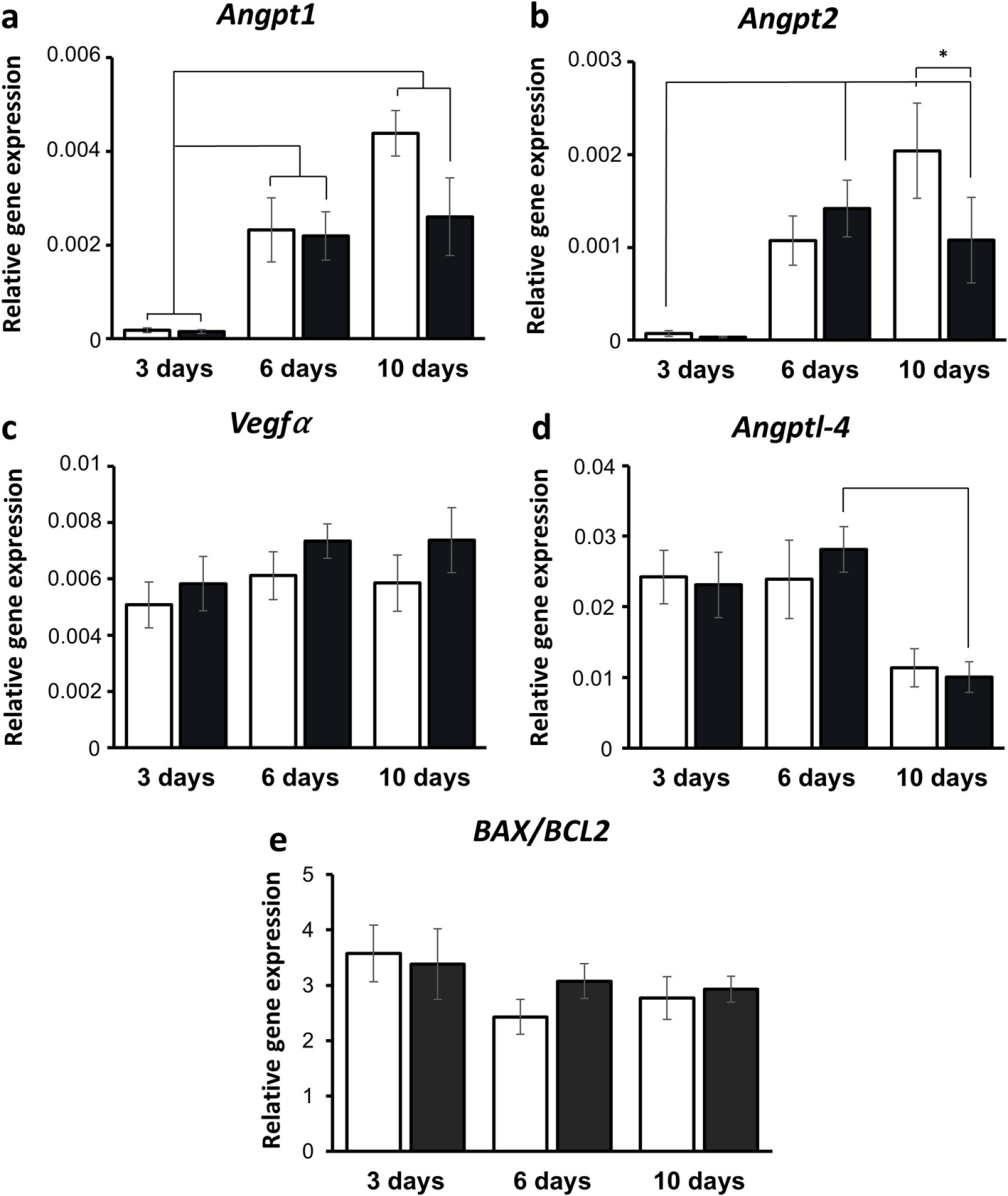
Expression of angiogenic and apoptotic genes in human ovarian xenografts after 3, 6 and 10 days. (**a-d**) Relative expression of the murine angiogenic genes: Angiopoetin 1 (*Ang1*); Angiopoetin 2 (*Ang2*); vascular endothelial growth factor alpha (*Vegfα*); Angiopoetin-like-4 (*Angptl4*). (**e**) Ratio of human anti- and- pro- apoptotic factors B-cell lymphoma2 (*BCL2*) and Bcl-2-associated X protein (*BAX*). Data presented as (mean ± SEM), and significant differences between timepoints are indicated by lines and symbols represent significant treatment effect

Gene expression of angiogenic and apoptotic markers was evaluated in human ovarian tissue after 3, 6 and 10 days xenografting. The expression of murine *Ang1* and *Ang2* had similar patterns of expression over the different time points for both the needle and control group (Fig. 5a+ b). In the control group both genes increased from day 3, to day 6 and to day 10, however, in the needle group the expression of *Ang1* and *Ang2* did not increase from day 6 to day 10 but remained at the level of day 6 (*p* < 0.001, *p* = 0.027). The gene expression of murine *Vegfα* showed a tendency toward a higher expression in the needle group compared to the control group at all three timepoints (Fig. 5c), and for day 6 this difference was almost significant (*p* = 0.051). The expression of murine *Angptl4* was similar on day 3 and 6 (Fig. 5d), but there was a decrease from day 6 to day 10 in both control and needle groups and the decrease was significant for the needle group (*p* = 0.043). The ratio of human *BAX/BCL2* was similar at all three time points and there was no difference between the needle and control groups (Fig. 5e).

### Follicle density and morphology

**Fig. 6:**
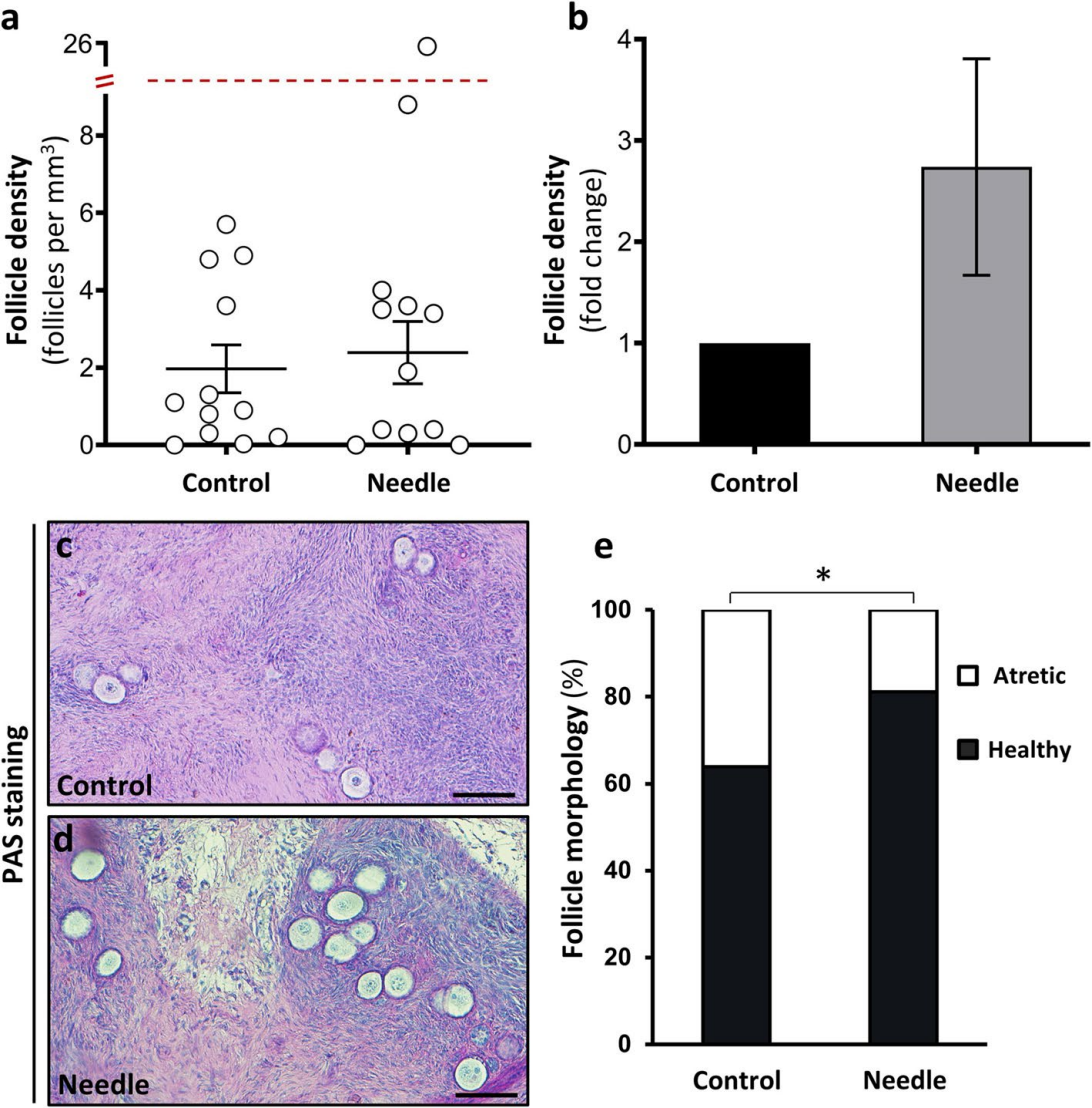
Follicle density and morphology in human ovarian xenografts after 4 weeks. (**a**) Follicle density for control and needle group after 4-week xenografting. Each dot represents a patient, and values are shown as number follicles per mm^3^ (mean ± SEM). (**b**) Follicle density given in fold change within paired samples from the same patient. (**c**, **d**) PAS-staining showing representative examples of follicles (scale bar = 100 µm) in the control (**c**) and needle group (**d**). (**e**) Follicle morphology shown in percentage of total number of follicles. Symbol represent significant treatment effect

The xenografted ovarian tissue retrieved after 4 weeks was used to evaluate the morphology and the total number of follicles in each graft (Fig. 6). A total of 1150 follicles were counted. Based on the follicle number and the volume of the graft, the follicle densities were estimated (Fig. 6a). The mean follicle density in the xenografted needle treated tissue was 5.18 follicles/mm^3^ ± 2.24 with a range of 0.3 to 26.0 follicles/mm^3^, compared to a density of 2.36 follicles/mm^3^ ± 0.67 with a range of 0.3 to 5.7 follicles/mm^3^ in the xenografted controls. Moreover, 7 out of 12 patients had a higher follicle density in the needle group compared to the control. These results showed a 1.7-fold higher follicle density for the treatment group compared to the grafted controls, however this difference was not significant (*p* = 0.208) (Fig. 6b). There was no significant difference between follicle stages in the two groups, with 93–97% of all follicles counted being primordial follicles. After morphological assessment, 292 out of the total 1150 follicles were categorized as atretic. For the needle group 19% of the follicles was atretic and for the grafted control it was 36% (Fig. 6e), and this difference was significant (*p* = 0.045).

## Discussion

This is the first study investigating if mechanical injury by needle puncturing could increase revascularization and hereby follicle survival in xenotransplanted human ovarian tissue. Current results showed no statistically significant differences in murine vessel density in human ovarian xenografts after needle puncturing compared to untreated control grafts. However, gene expression data did show differences in the expression patterns of angiogenic factors indicating an effect of the needle puncturing on the regulation of these genes. Furthermore, a higher follicle survival was observed in the needle group compared to control, although not statistically significant, and there was a significant difference in morphology, where the needle group had more morphological healthy follicles compared to control. Taken together our findings indicate that needle puncturing does not directly increase revascularization of ovarian xenografts, but it appears to affect the expression of angiogenic genes and has a positive effect on the follicle morphology.

Without vascular re-anastomosis, the tissue grafted is solely dependent on the development and invasion of new blood vessels. Xenografting studies has previously shown that the host is primarily responsible for revascularization of the xenografted human ovarian cortex [49]. And research in rodents has shown that the first signs of revascularization is observed two to three days after transplantation and stabilization is achieved after a little more than a week [49–51]. As expected, we observed very little murine vessel formation on day 3, confirming that this time point shows the initial vessel formation, this also correlates with another study which could not measure the partial pressure of O_2_ before day 3 in xenografted human ovarian tissue [52]. After 6 days we observed an increase in vessel formation compared to day 3 xenografts, however, the observed increase was not significantly higher. Between day 6 and 10 we observed a significant increase in vessel density in the human ovarian xenografts, reaching a vascularized area of approximately 3% of the grafts, which is comparable with our previous study [21]. These results show that the main vascularization of the grafted ovarian tissue occurs between day 6 and 10, confirming that areas of the graft are subjected to prolonged ischemia and hypoxia lasting up to at least 6 days. These results together with our previous findings [21] indicate that the burst in vessel formation occurs after 6 to 10 days, showing that the burst in vascularization happens during a short window of two to three days, giving us insight at which time point the ischemia/ reperfusion injury is the most intense. Furthermore, current results did not show any difference in vessel density between the needle group and control group, and we therefore conclude that needle puncturing did not increase revascularization of xenografted human ovarian tissue. In our previous study we used Er:YAG laser to induce controlled tissue damage in human ovarian cortex prior to xenotransplantation, and we found a significant decrease in revascularization and follicle survival compared to control after grafting which indicated that this form of tissue damage was detrimental to the graft [21]. Our current results using needle puncturing showed no signs of detrimental effects on revascularization, follicle survival or the ratio of *BAX/BCL2*, demonstrating that this is not a harmful procedure.

The gene expression data of *Vegf*, did show a tendency toward a treatment effect on day 6, and there was a higher expression level in the needle group compared to control at the other time points. Moreover, there was a consistent and similar expression on all time points, showing that *Vegfα* is expressed in the human ovarian tissue early and consistently after transplantation. The expression of *Ang1* and *Ang2* had similar patterns, which was surprising due to their opposite nature. ANG1 and ANG2 act through the same tyrosine kinase receptor, Tie-2. They both have pro-angiogenic effects in the presence of VEGF, however they exert opposite effects upon receptor binding. ANG1 stabilizes the blood vessels formed through the actions of VEGF [53], whereas ANG2 destabilizes and in the presence of VEGF is important for endothelial cell proliferation and migration [54]. If VEGF is not present ANG2 has antagonizing effects and leads to vessel destabilization followed by regression and endothelial cell death [55–57]. Moreover, ANG1 and ANG2 is an intricate and complex balance important for angiogenesis and has been shown to be important for follicular angiogenesis, ovulation, and corpus luteum formation [58]. The observed increase of *Ang1* and *Ang2* could be explained by the increase in angiogenic processes, however, *Ang2* is also increased and important during inflammation and hypoxia [59–61], which could explain the increase of *Ang2* expression from day 3, 6 and also day 10 for the control group. However, the same increase was not observed for the needle group, which is consistent with our previous results using the Er:YAG laser [21]. ANGPTL4 has been proposed to have the same context dependent pro- and anti-angiogenic effect as ANG2 [62–64], and also having the same co-dependent relationship with VEGF to be proangiogenic [65]. Furthermore, ANGPTL4 is important for wound healing and expression is stimulated by hypoxia [64, 66]. *Angptl4* was similarly expressed on day 3 and 6, but there was observed a decrease for both groups from day 6 to day 10, which could indicate the first pro-angiogenic period, where wound healing and revascularization is occurring stimulated by the hypoxic period, where on day 10 there may be a stabilization which leads to the decrease in *Angptl4* expression. Taken together, the results indicate that the needle puncturing did affect the expression of angiogenic genes, and it illustrates the complexity of the revascularization process.

Current results showed significantly fewer atretic follicles and a tendency toward a higher follicle density in the needle group compared to the control group. Moreover, no signs of follicle activation were observed in the needle punctured tissue as the vast majority of follicles were primordial follicles (97%) which was similar to the control group (93%). These results is comparable to our previous study where the Er:YAG laser treatment resulted in a lower follicle survival, however, the proportion of follicles in the different stages did not differ and did not suggest follicle activation in the laser treated group compared to control [21]. The effect on the follicle survival cannot be explained by a higher revascularization of the tissue, however we did observe an effect on the angiogenic genes, showing that the needle puncturing did affect the gene expression in the xenografted tissue. Therefore, it is plausible that other signaling pathways associated with wound healing can be affected by the needle puncturing and contribute to the beneficial effects on follicle survival. Furthermore, controlled tissue damage in the form of needle puncturing can to some degree be compared with tissue fragmentation, because both methods inflict mechanical injury to the tissue. De Roo and colleagues' found that fragmentation of human ovarian tissue before culture resulted in activation of primordial follicles through the hippo and PI3K/Akt pathway [67]. Furthermore, ovarian fragmentation has been studied as a way to activate follicle growth as treatment for premature ovarian insufficiency [68]. However, a study by Lunding and colleagues did not find the same effect of fragmentation when human ovarian tissue was transplanted to immunodeficient mice for 6 weeks as no significant differences in follicle stages were reported [69]. These studies demonstrate that there are contradicting results regarding follicle activation following ovarian tissue fragmentation or disruption. Taken together, there is evidence that the way the mechanical injury is performed influences the outcome, and that is also varies between tissue types [21, 42, 70]. In this study, the mechanisms behind the positive effects on follicle survival and morphology induced by the needle puncturing cannot be explained by increased revascularization which warrants further investigations to uncover potential mechanisms and to investigate the possibilities with mechanical injury.

## Conclusion

Needle puncturing of thawed human ovarian tissue prior to transplantation was not able to increase revascularization in the human ovarian xenografts. However, needle puncturing did affect the expression of angiogenic genes and resulted in slightly higher follicle survival and significantly better follicle morphology in the ovarian xenografts compared to control. Current results call for further investigations of needle puncturing in the context of OTT.

## Data Availability

The datasets used and/or analysed during the current study are available from the corresponding author on reasonable request.
